# Systematic review and meta-analysis of the discriminatory performance of risk prediction rules in febrile neutropaenic episodes in children and young people

**DOI:** 10.1016/j.ejca.2010.05.024

**Published:** 2010-11

**Authors:** Bob Phillips, Ros Wade, Lesley A. Stewart, Alex J. Sutton

**Affiliations:** a Centre for Reviews and Dissemination, University of York, York YO10 5DD, United Kingdom; b Department of Health Sciences, University of Leicester, Leicester LE1 7RH, United Kingdom

**Keywords:** Systematic review, Meta-analysis, Neutropaenic sepsis, Clinical decision rules, Diagnosis

## Abstract

**Introduction:**

Febrile neutropaenia is a frequently occurring and occasionally life-threatening complication of treatment for childhood cancer, yet many children are aggressively over-treated. We aimed to undertake a systematic review and meta-analysis to summarise evidence on the discriminatory ability and predictive accuracy of clinical decision rules (CDR) of risk stratification in febrile neutropaenic episodes.

**Methods:**

The review was conducted in accordance with Centre for Reviews and Dissemination methods, using random effects models to undertake meta-analysis. It was registered with the HTA Registry of systematic reviews, CRD32009100453.

**Results:**

We found 20 studies describing 16 different CDR assessed in 8388 episodes of FNP. No study compared different approaches and only one CDR had been subject to testing across multiple datasets. This review cannot conclude that any system is more effective or reliable than any other.

**Conclusion:**

To maximise the value of the information already collected by these and other cohorts of children with febrile neutropaenia, an individual-patient-data (IPD) meta-analysis is required to develop and test new and existing CDR to improve stratification and optimise therapy.

## 1 Introduction

Children undergoing treatment for malignancy have an excellent chance of survival, with overall rates approaching 75%.^1^ In most cases, children who die following treatment for cancer do so of their disease, but despite huge improvements in supportive care, around 16% of deaths within 5 years of diagnosis are due to the complications of therapy.^2,3^ One such life-threatening complication in immunocompromised children remains infection, frequently presenting as the occurrence of fever with neutropaenia.^4^ A robust risk stratification model which reliably predicted those children at very low risk of having a significant infection could result in reduced intensity and/or duration of hospitalised antibiotic therapy. Those at high risk of complications could be targeted for more aggressive management. At present there are many differing policies for the management of febrile neutropaenia in paediatric practice^5,6^ with lack of agreement about how and which clinical decision rules (CDR), if any, are used.

A clinical decision rule is a tool designed to be used at the bedside to assist clinical decision making.^7^ These rules should be validated by assessing them on a separate population; to test both how well the rule differentiates the risk groups (discriminatory ability) and to determine the absolute estimates of risk within these groups (predictive accuracy).

In adult oncology practice the Multinational Association for Supportive Care in Cancer (MASCC) risk index^8^ provides a CDR to identify patients at low risk of serious medical complications during febrile neutropaenia. The factors identified included ‘young age’ (<60 years) and no chronic obstructive airways disease, among other features specific to the disease type and presentation at each episode, and has been used as the basis for the out-patient management of fever in low-risk neutropaenic adult patients.^9^ The MASCC rule is of very limited applicability in this group: it did not include children in its derivation, the age criterion is non-discriminatory, and chronic airways disease is extremely rare. Accordingly, studies on children and young people require separate, detailed examination.

This systematic review aimed to identify, critically appraise and synthesise evidence on the discriminatory ability and predictive accuracy of existing CDRs in febrile neutropaenic episodes in children and young people undergoing treatment for malignant disease.

## 2 Materials and methods

The review was conducted in accordance with ‘Systematic reviews: CRD’s guidance for undertaking reviews in health care^10^ and registered on the HTA Registry of systematic reviews: CRD32009100453. It sought studies which aimed to derive or validate a CDR in children or young people (aged 0–18 years) presenting with febrile neutropaenia. Both prospective and retrospective cohorts were included, but those using a case–control (‘two-gate’) approach were excluded as these have been previously shown to exaggerate diagnostic accuracy estimates.^11^ Studies exclusively addressing the prediction of radiologically confirmed pneumonia are subject to a separate review [in submission to Arch Dis Child – Still under consideration].

### 2.1 Search strategy and selection criteria

An electronic search strategy (see Web Appendix 1) was developed which examined the following databases from their inception to February 2009:

• MEDLINE
• MEDLINE In-Process and Other Non-Indexed Citations
• EMBASE
• CINAHL
• Cochrane Database of Systematic Reviews (CDSR)
• Database of Abstracts of Reviews of Effects (DARE)
• Health Technology Assessment Database (HTA)
• Cochrane Central Register of Controlled Trials (CENTRAL)
• Conference Proceedings Citation Index - Science (CPCI-S)
• Literatura Latinoamericana y del Caribe en Ciencias de la Salud (LILACS)

Reference lists of relevant systematic reviews and included articles were reviewed for further relevant articles. Published and unpublished studies were sought and no language restrictions applied. Non-English language studies were translated. Two reviewers independently screened the title and abstract of studies for inclusion, and then the full text of retrieved articles. Disagreements were resolved by consensus.

### 2.2 Validity assessment and data extraction

The validity of each study was assessed using 11 of the 14 questions from the QUADAS assessment tool for diagnostic accuracy studies.^12^ The QUADAS tool was adapted specifically for the review,^13^ omitting questions on ‘time between index and reference test’, ‘intermediate results’ and ‘explanation of withdrawals’ (see Web Appendix 2). The CDR and reference tests are necessarily related, and the design of a CDR means that ‘intermediate’ results are included in any analysis. The issue of incomplete data was addressed in the analysis of the method of derivation or validation, and as such was not included as a quality criterion.

Data were extracted by one reviewer and checked by the other. The data extracted included age and sex distribution of the included participants, geographical location of the study and participant inclusion/exclusion criteria. The performance of the CDR as a 2 * *k* table (where *k* refers to the number of strata described) as well as the methods used to derive the CDR (where applicable), the variables considered, methods of statistical analysis and approach to multiple episodes in individual patients and missing data were also extracted.

### 2.3 Methods of analysis/synthesis

Quantitative synthesis was undertaken for studies which tested the same CDR and, where appropriate, was investigated for sources for heterogeneity.

For dichotomous test data, analyses were attempted with a bivariate model (using ‘metandi’ in STATA10^14^). For tests with very small numbers of studies to pool (*n* ⩽ 4) fitting a bivariate model is problematic as the procedure frequently fails to converge. In these cases, a univariate approach was used (pooling sensitivity and specificity separately).^15^

For tests where three-level (low, medium and high risk) results were produced, an approach based on a previous meta-analysis of three-level CDR results was used.^16^ This random-effects meta-analysis was undertaken using WinBUGS 1.4.3^17^ to estimate the proportions of individuals classified as low, medium or high risk in the bacteraemic and non-bacteraemic groups. As an extension to this method, bivariate random effects were applied to the calculation of each proportion. Data from studies which used a similar rule but provided only two of the risk categories (i.e. low versus medium–high) were also included in this analysis.^18^ These proportions were used to calculate likelihood ratios (LR) for each risk category and the corresponding 95% credible intervals (CrI).

Heterogeneity between study results was explored through consideration of study populations, study design, predictor variables assessed and outcomes chosen, although the small number of studies in each category limited this approach. Sensitivity analysis was undertaken by comparing results when the original (derivation) dataset was included and excluded.

For those areas where a quantitative synthesis was not possible, a narrative approach was used.

## 3 Results

**Fig. 1:**
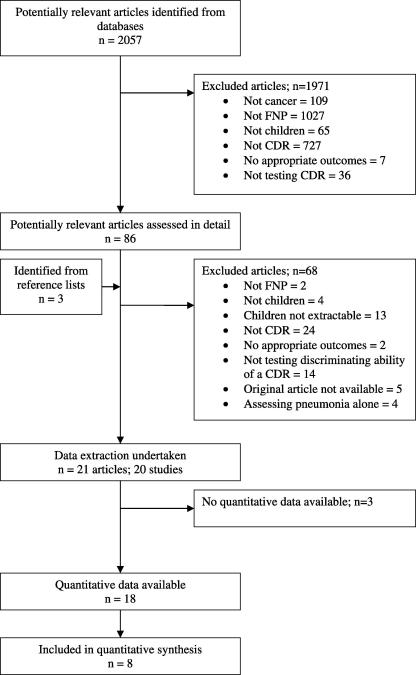
Flow diagram of study selection process.

Twenty-one articles reporting on 20 studies^19–38^ were eligible for inclusion in the review (see Fig. 1). The studies included patients from 1 month to 23 years old, with a wide range of malignancies, and a total of 7840 episodes of FNP describing 11 outcomes, summarised in 5 clusters: death, critical care requirement, serious medical complication, significant bacterial infection and bacteraemia (see Table 1). Eight of these studies were prospective,^25,26,29,30,32,33,36,37^ 11 were retrospective^19–23,27,28,31,34,35,38^ and 1 was a retrospective analysis of prospectively collected data.^24^

### 3.1 Quality assessment

The studies varied in quality. Potential biases due to threats to independent outcome assessment were present in some studies (see Web Appendix 3). The applicability of the studies to specific populations also varied (see Table 1). Thirteen definitions of febrile neutropaenia were used, with 12 definitions of fever and 4 of neutropaenia. However, all definitions are clinically similar, with any variation at the ‘lowest risk’ part of the spectrum of classification.

### 3.2 Techniques of CDR derivation

The 16 reports of attempts to derive a CDR varied in the populations included the predictor variables and adverse outcomes they reported. The model-building technique, the reporting and handling of missing data and multiple-episode data and the use and categorisation of continuous and categorical variables were also assessed. Details are available in Web Appendices 4 and 5.

### 3.3 CDR performance

**Fig. 2:**
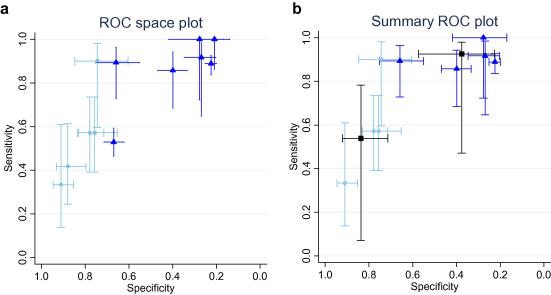
(a) Results of the individual ‘Rackoff’ CDR studies. The ROC space plots show each study estimates of sensitivity and specificity as a marker at the point estimate, with 95% confidence intervals demonstrated by lines. In reading such graphs, tests with a better discriminatory ability fall in the top left corner of the plot, and non-discriminatory tests fall on a 45° line between the bottom left and top right. The dashed lines (light blue)/circles represent the dichotomy of low and medium versus high risk groups (5 datasets), the solid lines (darker blue)/triangles between low versus medium and high (7 datasets). The outlier (32) is towards the centre of the graph. (b) Pooled results of the ‘Rackoff’ CDR meta-analysis. The dashed lines (light blue)/circles represent the dichotomy of low and medium versus high risk groups (4 datasets), the solid lines (darker blue)/triangles between low versus medium and high (5 datasets). The meta-analytic summary estimates are shown in heavy lines (black)/squares. (For interpretation of the references to colour in this figure legend, the reader is referred to the web version of this article.)

**Fig. 3:**
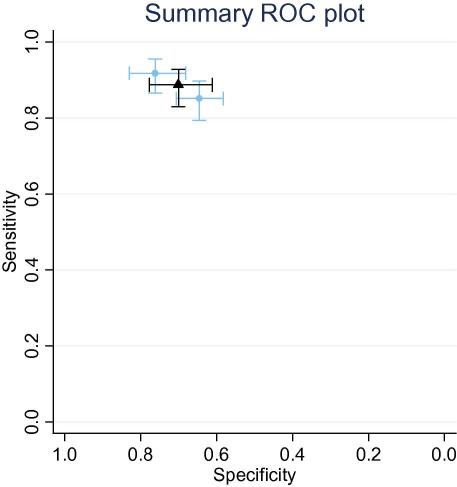
Pooled and individual results of the ‘Santolaya’ CDR studies. The dashed lines (light blue)/circles represent the individual studies. The meta-analytic summary estimates are shown in heavy lines (black)/squares. (For interpretation of the references to colour in this figure legend, the reader is referred to the web version of this article.)

The CDR had diverse test performance (see Table 2 for detail). This heterogeneity has largely been explored using a narrative structure, as pooling across all the studies was not possible due to the varied rules, outcomes and populations studied. It was examined by analysis of the tabulated CDR performance data and graphically with plots of sensitivity and specificity (Web Fig. 1 for unpooled studies and Figs. 2 and 3 for pooled studies).

Meta-analysis of studies which used identical CDR was undertaken in two cases: the ‘Rackoff rule’^36^ to examine bacteraemia,^22,26,28,34,36^ and the ‘Santolaya rule’ for serious infectious complications.^32,33^

The ‘Rackoff rule’ discriminates between three groups of individuals at low, moderate and high risk of bacteraemia. A study which reported ‘microbiologically documented infection’ rather than the narrower ‘bacteraemia’ appeared as a significant outlier (see Fig. 2a).^34^ Exclusion of this study led to a more Normal distribution of the posterior probability plots. Undertaking a sensitivity analysis by exclusion of the initial rule derivation study demonstrated poorer discriminatory ability (see Fig. 2b for a ‘best estimate’ summary) LR [low] = 0.22 (95% CrI 0.03–1.85), LR [medium] = 0.79 (95% CrI 0.12–2.06) and LR [high] = 3.41 (95% CrI 0.24–18.7). The probability of bacteraemia in each of these groups will vary with the baseline chance of bacteraemia. If we use a 22% overall prevalence of bacteraemia (the average proportion over the included studies which report these data) the predictive values are; Low risk = 6% (95% CrI 1–34%), Mid risk = 18% (CrI 3–37%) and High risk = 49% (95% CrI 6–84%).

The ‘Santolaya rule’ showed a moderate ability to differentiate between low- and high risk groups considering the outcome of ‘invasive bacterial infection’. The derivation sample performed marginally less effectively than the validation set. The pooled estimate of test accuracy is LR [low] = 0.17 (95% CI 0.12–0.23) and LR [high] = 2.87 (95% CI 2.43–3.38), see Fig. 3. Using the average ‘invasive bacterial infection’ rate of 47%, this leads to the probability of ‘invasive bacterial infection’ in the low group as 13% (95% CI 9–13%) and 72% (95% CI 68–75%) in the high group. The two studies examining this rule are from the same research group (although in a multi-centre study environment) and the rule has not been subject to further validation.

Assessments of potential sources of heterogeneity showed that derivation studies generally had better accuracy than validation studies. The outcome studied also appeared to alter rule performance but the heterogeneity of rules and populations make this difficult to examine clearly. Those CDRs developed in a population where the highest risk patients are excluded (e.g. bone marrow transplant recipients) did not seem to differ from rules developed without these exclusions. All these analyses are confounded by the correlation of location, population, outcome and rule. For example: the Santolaya studies took place in Chile, excluded BMT, looked at a broad definition of infectious complications and developed a 5-item rule, the Rackoff model was developed in the United States, did not clearly exclude any patient group, primarily examined bacteraemia and produced a rule based on a single haematological parameter and temperature.

Examination of the detailed content of all the proposed rules shows they address four major domains (Web Appendix 6). The first can be considered stable patient-related factors, including age and the underlying disease. The second group reflects treatment; the presence of a central venous catheter and the type or duration since last chemotherapy. The third group reflects episode-specific clinical features, such as maximum temperature, the patient’s blood pressure or clinical features of infection. The final group contains episode-specific laboratory test values. These are various markers of bone marrow function where, excepting,^23^ each rule uses a single item which reflects one of the three major cellular components: haemoglobin, platelets, leucocytes (or a subset); and serum inflammatory markers (C-reactive protein). An exploratory analysis of the individual features common across predictive studies shows that age, malignant disease state, clinical assessments of circulatory and respiratory compromise, higher temperatures and bone marrow suppression all have some explanatory power.

## 4 Discussion

This is to our knowledge the first systematic review and meta-analysis of risk prediction rules in paediatric febrile neutropaenia. It describes 20 studies producing 16 separate models, assessing a variety of outcomes, with individual differences in definitions, covering five main categories: death, critical care requirement, serious medical complication, significant bacterial infection and bacteraemia. Despite the inclusion of nearly 8000 episodes of FNP, this review cannot conclude that any system is more effective or reliable than any other.

A clinical decision rule for febrile neutropaenic episodes can be broadly considered to have two uses. Primarily it is to decide if the risk of an episode is ‘low enough’ to allow reduced intensity therapy (e.g. outpatient management), but at the opposite end of the risk scale, a CDR may be helpful to direct increasingly close observation and more aggressive management. The patients at ‘high risk’ do not have such clear management options: there are no effective truly prophylactic measures to prevent sepsis syndrome but early recognition may prevent progression to septic shock.^39^

The majority of CDR in this review focus upon defining a group at ‘low risk’ of complications. Two rules in particular have been subject to greater verification, other rules show promise and have clinical/physiological similarities, but have had less validation.

The performance of only one rule could be reasonably assessed across multiple datasets; that of absolute monocyte count and temperature criteria proposed by Rackoff^36^ to exclude bacteraemia. This CDR, tested in 1171 episodes over five datasets, in three different groups across time and in different centres, has the greatest strength of evidence. The most appropriate pooled estimate of the rule’s effectiveness shows limited discriminatory ability, LR [low] = 0.22 (95% CrI 0.03–1.85), LR [medium] = 0.79 (95% CrI 0.12–2.06) and LR [high] = 3.41 (95% CrI 0.24–18.7). The marked uncertainty in these estimates is best demonstrated by the post-test probabilities of bacteraemia: Low risk = 6% (95% CrI 1–34%), Mid risk = 18% (CrI 3–37%) and High risk = 49% (95% CrI 6–84%).

Of the other rules the Santolaya model^33^ shows a moderate ability to differentiate between groups at low and high risk of ‘serious infection’, but again with marked uncertainty (LR [low] = 0.17 (95% CI 0.12–0.23) and LR [high] = 2.87 (95% CI 2.43–3.38), post-test probabilities for the low risk group = 13% (95% CI 9–13%) and in the high risk group 72% (95% CI 68–75%)). The rule has been developed and tested in Chile, which may limit its applicability in Western Europe and North America. The proportion of patients with bacteraemia (∼25%) is similar to the other studies in this review, but their broad definition of adverse medical outcomes, as found in ∼50% of cases, does not have a direct comparator among the Western European/North American studies reviewed, therefore no accurate conclusion can be reached. This reflects an uncertainty in the selection of the desired and measurable outcome. An ideal study would consider not just death, critical care requirement, serious medical complication, significant bacterial infection or bacteraemia but ‘an absence of adverse consequences’.

Any adaptation or development of a new rule should primarily look to assess the variables shown in the many CDR reviewed to be of predictive value, over those found purely by ‘*p*-value’ sampling of bivariable testing. In addition to reducing random error, building upwards from the simple clinical variables of age, disease and basic clinical examination will ensure any complex tests add significant value to the fundamentals of patient assessment.

An analysis of the techniques used to build the CDR was incorporated into this review. The studies are spread across a number of years, and during that time there have been significant methodological developments and technological improvements which have made previously complex computation within the reach of many health researchers. However, a series of previously described methodological problems with diagnostic/prognostic model papers were present in this review. These included: small event-per-variable ratios leading to models more likely to be overfitted to their original dataset and disappointing in clinical practice^40^; overestimation of accuracy from derivation studies; failure to examine for non-linear relationships, which may misjudge a predictor as unimportant,^41^ for example, there are plausible reasons to assume that patient age may have a non-linear ‘U’-shaped relationship with infection and outcome,^42^ as should time-from-chemotherapy; use of data-driven stepwise variable selection and cutpoint determination techniques which may give spurious results^43,44^; premature categorisation of continuous data; lack of examination of missing data and suboptimal examination of clustered data.

This review has demonstrated a wide range of rules for the prediction of adverse outcomes during episodes of febrile neutropaenia in children. None of the rules identified has been subject to the extensive geographical and temporal discriminatory validity assessments that mark the highest quality CDRs, and many potential difficulties with model building have been identified. Practical application of many of these CDR within an in-patient environment is likely to be safe but without further research uncertainty will remain as to the efficiency of the CDR in use. To provide this information and maximise the value of the information already collected by these and other cohorts of children with febrile neutropaenia, an individual-patient-data (IPD) meta-analysis is being undertaken to develop and test new or existing prediction models and provide a firmer basis for stratified treatment trials in this common and occasionally fatal complication of therapy.^45^

## Contributions

R.S.P. conceived the idea and co-ordinated and led the review, developed the protocol, undertook screening and data extraction, synthesis and drafted the manuscript. He has had full access to the data and has final responsibility for the paper and the decision to submit. R.W. developed the protocol, undertook screening and data extraction and read and modified the manuscript. L.A.S. developed the protocol, reviewed the results and read and modified the manuscript. A.J.S. developed and checked the synthesis and read and modified the manuscript.

## Funding

R.S.P. is supported by an MRC Research Training Fellowship G0800472, which also supported R.W. for this review. A.J.S. and L.A.S. received no external funding for their work in this study. The funder had no role in the design or conduct of the study nor the production of, or decision to submit, this manuscript.

## Conflict of interest statement

None declared.

## Figures and Tables

**Table 1:** Studies of clinical decision rules.

Citation	Derivation or validation study	Study location (years)	Inclusion criteria	Exclusion criteria	Total number of patients	Total number of episodes	Age of patients	Clinical prediction rule^a^	Outcomes
Adcock (1999)	Derivation of CDR	North Carolina, USA (1995–1996)	ANC <1000 cells/mm^3^, temperature ⩾38 °C, HIV −ve		33	88	Median 5 y (range 1–18 y)	High risk = hypotension/septic shock, inflamed central line site, recent high dose Ara-C	Gram-positive bacteraemia
Alexander (2002)	Derivation of CDR	Boston, USA (1994–1995)	ANC ⩽500/mm^3^, temperature >38.5 °C. Outpatient status	Post stem cell transplant	104	188	Mean 8.9 y (SD 5.7 y)	Low risk = not AML/Burkitts/Induction ALL/Progressive-relapsed with marrow involvement (‘Anticipated neutropaenia <7 d’) and no significant comorbidity (defined as hypotension, tachypnea/hypoxia <94%, new CXR changes, altered mental status, severe mucositis, vomiting or abdominal pain, focal infection, other clinical reason for in-patient treatment)	Bacteraemia
									Serious medical complication Death
								High risk = hypotension/septic shock, inflamed central line site, recent high dose Ara-C	
Ammann (2003)	Derivation of CDR	Berne, Switzerland (1993–2001)	ANC ⩽500 cells/mm^3^ or ⩽1000 cells/mm^3^ and falling, axilliary temperature ⩾38.5 °C for ⩾2 h, or once ⩾39 °C	Patients with FN due to malignant bone marrow suppression, or following myeloablative therapy	132	364	Not reported	Low risk = not AML/Burkitts/Induction ALL/Progressive-relapsed with marrow involvement (‘Anticipated neutropaenia <7 d’) and no significant comorbidity (defined as hypotension, tachypnea/hypoxia <94%, new CXR changes, altered mental status, severe mucositis, vomiting or abdominal pain, focal infection, other clinical reason for in-patient treatment)	Severe bacterial infection, (death from bacterial infection, a positive culture of normally sterile body fluids, radiologically proven pneumonia, clinically unequivocal diagnosis of a bacterial infection, or CRP > 150 mg/L)
(regression models)								Low risk ⩽ 3 factors (model #2) or ⩽4 factors (model #3). Risk factors = bone marrow involvement, absence of clinical signs of viral infection, high serum CRP level, low leucocyte count, presence of a central venous catheter, high haemoglobin level, and Pre-B-cell leukaemia	Severe bacterial infection (death from bacterial infection, a positive culture of normally sterile body fluids, radiologically proven pneumonia, clinically unequivocal diagnosis of a bacterial infection, or CRP > 150 mg/L)
Ammann (2004) (same population as Ammann (2003))	Derivation of CDR			Additionally, patients with established severe bacterial infection	111	285	Median 6.3 y (interquartile range 3.2–12.1 y)	Low risk = all of: maximum temp ⩽ 39.7 °C, no comorbidity requiring hospitalisation, leucocyte count > 0.5 × 10^9^/L, and in partial or complete remission	Bacteraemia
Gala-Peralta (2005)	Validation of CDR	Barcelona, Spain (2002)	ANC ⩽ 500/mm^3^, ‘fever’ (temperature not defined)		30	62	Mean 8.7 y (range 1.2–14.7 y)	Low risk ⩽ 2 of: <1 y, poor bone marrow response (plt < 75, ANC < 100),uncontrolled solid tumour or relapsed leukaemia, chemotherapy <10 d earlier, rapid neutropaenia, cardiac and renal dysfunction	Positive blood culture
Hann (1997)	Derivation of CDR	Multiple centres across Western Europe (1986–1994)	ANC ⩽1000 cells/mm^3^, temperature ⩾ 38.0 °C twice in <12 h, or once ⩾ 38.5 °C, in an EORTC trial		759	759	Median 8 y	No rule described	Bacteraemia
								Individual features = disease type, IV line, shock, duration of granulocytopaenia and admission temperature	
Jones (1996)	Derivation of CDR	North Carolina, USA (1987–1993)	ANC <500 cells/mm^3^, oral temperature ⩾ 38.0 °C ⩾12 h, or once >38.5 °C	None reported, but ‘none of the children were undergoing BMT’	127	276	Mean 8 y (range 2 m to 21 y)	Low risk = ANC ⩾ 200, outpatient at onset, in remission	Bacteraemia Clinical infection
Lucas (1996)	Derivation of CDR	New York, USA (1990–1992)	ANC <500 cells/mm^3^ or <1000 cells/mm^3^ and falling, temperature ⩾ 38.0 °C ⩾2 occasions in ⩾12 h, or once ⩾38.5 °C. Outpatient status	Received blood product transfusions within 6 h or cytosine arabinoside within 2 d of presentation	161	509	Mean 9.2 y (range 1–18 y)	Low risk = no chills, hypotension, or a requirement for fluid resuscitation at admission	Positive blood culture ICU admission Septic death
Petrelli (1991)	Validation of CDR	Camargo, Brazil (1988–1989)	ANC ⩽ 500 cells/mm^3^, temperature ⩾ 37.5 °C ⩾3 occasions in ⩾24 h, or once ⩾38.0 °C. Outpatient status	Fever associated with blood product transfusions or drugs	146	240	Mean 7.3 y	Low risk: patients with solid tumours and lymphomas stages I and II. High risk: patients with leukaemias and lymphomas stages III and IV	Positive blood culture
Riikonen (1993)	Derivation of CDR	Helsinki, Finland (1989–1990)	ANC < 200 cells/mm^3^, temperature >38.0 °C ⩾2 occasions in ⩾4 h, or once >39.0 °C	Antibiotics (excluding Septrin) in the preceding 3 weeks	46	91	Range 1–16 y	No rule described. No variables emerged as significant	Bacteraemia Focal infection Suspected sepsis/fever of unknown origin
Rojo (2008)	Derivation of CDR	Santiago, Chile (2003 –2006)	Episode of febrile neutropaenia which was ‘low risk’ according to the PINDA criteria		33	47	Median 5.8 y (1.1–15.7 y)	No rule described. No variables emerged as significant	‘Unfavourable outcome’ – Compound of: haemodynamic instability, new focus if bacterial infection, 72 h persistent fever, unresponsive CRP, or continuing +ve blood cultures 72 h after treatment
Rondinelli (2006)	Derivation of CDR	San Paulo, Brazil (2000– 2003)	ANC < 500 cells/mm^3^ or ⩽1000 cells/mm^3^ and falling, temperature ⩾37.8 °C ⩾3 occasions in ⩾24 h, or once >38.0 °C. First episode per patient (new or relapsed disease)	Second or subsequent episode. Episodes in progressive disease (<6 m from between completing therapy and relapse). History of BMT	283	283	Mean 5.2 y	Low risk = 2.5–5 points: Intermediate risk = 5.5– 9 points: High risk = Greater than 9 points. 4.5 points for: clinical site of infection; 2.5 points for: no URTI; 2 points for: CVC; 1 point for: aged ⩽ 5 y, fever > 38.5 °C, Hb ⩽7 g/dL	‘Serious infectious complication’ – sepsis, shock, +ve blood cultures, infection-related death
Tezcan (2006)	Derivation of CDR	Antalya, Turkey (1996–2004)	ANC < 500 cells/mm^3^ or <1000 cells/mm^3^ and falling, axilliary temperature ⩾38.0 °C ⩾2 occasions at 4 h intervals, or once >38.3 °C	Fever that occurred following transfusion of blood and blood products or administration of G-CSF	240	621	Median 6 y (range 1 m to 17 y)	No rule described. Significant association between hypotension, uncontrolled cancer and mortality. Duration of fever only independent risk factor for microbiologically documented infection	Death Clinically suspected infection Microbiologically documented infection
West (2004) (internally validated using bootstrap)	Derivation of CDR	California, USA (1994–1998)	ANC < 500 cells/mm^3^ or <1000 cells/mm^3^ and falling, axilliary temperature ⩾38.0 °C ⩾3 occasions in 24 h, or once ⩾38.5 °C, within 21 d of chemotherapy	Induction, relapse and refractory disease. Collapse within 1 h of admission	143	303	Mean 7.6 y (SD 4.6 y)	Very high risk = temp > 39.5 °C and CRT > 3 s; High risk = temp > 39.5 °C or CRT > 3s; Low risk = neither	Requirement for critical care within 24 hs of presentation (fluid boluses ⩾60 ml/kg, inotropes or ventilation)

*Paganini rule*									
Paganini (2007)	Derivation	Multiple centres across Argentina (derived 1 institution, validated in 7 further ones) (2000–2004)	ANC < 500 cells/mm^3^ or <1000 cells/mm^3^ and falling, temperature ⩾ 38.0 °C ⩾2 occasions in 24 h, or once ⩾38.5 °C	History of BMT	458	714	Mean 7 y (range 1 m to 17.9 y: derivation set)	Low risk < 4. Mid risk = 4. High risk ⩾ 4. Advanced stage of disease = 3 points, Comorbidity = 2 points, Bacteraemia = 1 point	Death
	and validation of ‘Paganini rule’				523	806	Mean 7.1 y (range 1 m to 17.5 y: validation set)		

*Rackoff rule*									
Rackoff (1996)	Derivation of ‘Rackoff rule’	Indianapolis, USA (1994–1995)	ANC < 500 cells/mm^3^, temperature >38.0 °C ⩾3 occasions in ⩾ 24 h, or once >38.5 °C. Outpatient status		72	115	Range 9 ms to 18 y: derivation set	Low risk = AMC > 100; Mid risk = AMC < 100, and temp < 39; High risk = AMC < 100, but temp ⩾ 39	Bacteraemia Clinical reason for admission
Rackoff (1996)	Revision of ‘Rackoff rule’	(1993)				57	Validation set not reported	Low risk = AMC > 100	
Baorto (2001)	Validation/recalibration of ‘revised Rackoff rule’	St. Louis, Dallas and Houston, USA (1990–1996)	ANC < 500 cells/mm^3^, temperature ⩾ 38 °C, 12 m or older	History of BMT	558	1171	Mean 8.0 y (range 1–23 y)	Low risk = AMC > 100	Bacteraemia ICU/Death related to bacteraemia within 72 h of admission for FN
Klaassen (2000)	Derivation	Toronto, Canada (1996–1998)	ANC < 500 cells/mm^3^ or ⩽1000 cells/mm^3^ and falling. Temperature > 38.0 °C ⩾2 occasions in ⩾ 12 h, or once >38.5 °C, or localised infection	New malignant diagnosis; bone marrow or stem-cell transplantation in preceding 6 m. Another medical condition that independently required inpatient observation. Interstitial infiltrate or lobar consolidation on chest X-ray	140	227	Median 6.8 y (range 6 m to 17 y: derivation set)	Low risk = AMC > 100; Mid risk = AMC < 100, and temp ⩽ 39; High risk = AMC < 100, but temp > 39	Bacteraemia Significant bacterial infection (defined as any blood or urine culture positive for bacteria, interstitial or lobar consolidation on CXR, or unexpected death from infection before ANC recovery (>0.5 × 10^9^/L))
	and validation of CDR (‘Rackoff rule’)			Unclear	Unclear	136	Median 7.6 y (range 1–18 y: validation set)		
Madsen (2002)	Validation/recalibration of ‘Rackoff rule’	Indianapolis, USA (1997)	New admissions ‘coded’ as ‘fever of unknown origin’ and ANC < 500 cells/mm^3^	History of BMT. AML. In-patient status	76	157	Mean 8 y (range 2 m to 18 y)	Low risk = AMC > 100; Mid risk = AMC < 100, and temp < 39; High risk = AMC < 100, but temp ⩾ 39	Positive blood culture
Tezcan (2006)	Validation of ‘Rackoff rule’	Antalya, Turkey (1996–2004)	ANC ⩽ 500 cells/mm^3^ or ⩽1000 cells/mm^3^ and falling, axilliary temperature ⩾38.0 °C ⩾2 occasions at 4 h intervals, or once >38.3 °C	Fever that occurred following transfusion of blood and blood products or administration of G-CSF	240	621	Median 6 y (range 2 m to 17 y)	Low risk = AMC > 100	Death Clinically suspected infection Microbiologically documented infection
*Santolaya rule*									
Santolaya (2001)	Derivation of ‘Santolaya rule’	5 centres in Santiago, Chile (1996–1997)	ANC ⩽ 500 cells/mm^3^, axilliary temperature ⩾38.0 °C ⩾ 2 occasions 1 h apart, or once ⩾38.5 °C	Not reported	257	447	Mean 7 y (range 6 m to 18 y)	Low risk = 0 factors or isolated low plts or <7 d from chemotherapy. High risk = >1 risk factor, or isolated high CRP, hypotension or relapsed leukaemia. Risk factors: CRP ⩾ 90, hypotension, relapsed leukaemia, plts ⩽50, chemotherapy within 7 d	Invasive bacterial infection (positive blood culture – 2 for CoNS, positive bacterial culture from usually sterile site, or sepsis syndrome and/or focal organ involvement and haemodynamic instability and severe malaise) Death
Santolaya (2002)	Validation of ‘Santolaya rule’	6 centres in Santiago, Chile (1999–2000)	ANC ⩽ 500 cells/mm^3^, axilliary temperature ⩾38.0 °C ⩾ 2 occasions 1 h apart, or once ⩾38.5 °C	Not reported	170	263	Mean 7 y (range 7 m to 17 y)	Low risk = 0 factors or isolated low plts or <7 d from chemotherapy. High risk = >1 risk factor, or isolated high CRP, hypotension or relapsed leukaemia. Risk factors: CRP ⩾90, hypotension, relapsed leukaemia, plts ⩽ 50, chemotherapy within 7 d	Invasive bacterial infection (positive blood culture – 2 for CoNS, positive bacterial culture from usually sterile site, or sepsis syndrome and/or focal organ involvement and haemodynamic instability and severe malaise)

Age: y, years; m, months. ALL, acute lymphoblastic leukaemia; AML, acute myeloid leukaemia; AMC, absolute monocyte count; ANC, absolute neutrophil count; BMT, bone marrow transplant; CoNS, coagulase-negative *Staphylococcus*; CRP, C-reactive protein; CRT, capillary refill time; CXR, chest X-ray; Hb, haemoglobin; HIV, human immunodeficiency virus; Plt, platelets.

a Unless stated, the rule dichotomises into low and high risk groups.

**Table 2:** Predictive performance of clinical decision rules.

Citation	Clinical prediction rule	Number in study	Outcome	Number with outcome	Predictive accuracy		
					% Low	LR Low	LR High
*Models with one supporting dataset*							
Adcock (1999)	High risk = hypotension/septic shock, inflamed central line site, recent high dose Ara-C	33	Gram-positive bacteraemia	6		Data not given	
Alexander (2002)	Low risk = Nnt AML/Burkitts/Induction ALL/Progressive-relapsed with marrow involvement and no significant comorbidity	104	Bacteraemia	13	58%∗	0.24	2.39
Ammann (2003)							
(model #1: bootstrapped)	Final decision tree model: four covariates were used to classify low risk; bone marrow involvement, leucocyte count > 0.5 × 10^9^/L, with clinical signs of a viral infection, and aged up to 6 years at presentation. For those with a leucocyte count ⩽ 0.5 × 10^9^/L, they were further classified according to CRP level (⩽ or >50 mg/L)	111	Severe bacterial infection, (death from bacterial infection, a positive culture of normally sterile body fluids, radiologically proven pneumonia, clinically unequivocal diagnosis of a bacterial infection, or CRP > 150 mg/L)	90	10%	0	1.18
(model #2)	Low risk ⩽ 3 factors. Risk factors = bone marrow involvement, absence of clinical signs of viral infection, high serum CRP level, low leucocyte count, presence of a central venous catheter, high haemoglobin level, and Pre-B-cell leukaemia	(111)	As above	(90)	14%	0	1.29
(model #3)	Low risk ⩽ 4 factors. Risk factors = bone marrow involvement, absence of clinical signs of viral infection, high serum CRP level, low leucocyte count, presence of a central venous catheter, high haemoglobin level, and Pre-B-cell leukaemia	(111)	As above	(90)	20%	0.07	1.39
Ammann (2004)	Low risk = all of: maximum temp ⩽ 39.7 °C, no comorbidity requiring hospitalisation, leucocyte count > 0.5 × 10^9^/L, and in partial or complete remission	132	Bacteraemia	85	26%	0.15	1.40
Gala-Peralta (2005)	Low risk ⩽ 2 of: <1 year, poor bone marrow response (plt < 75, ANC < 100),uncontrolled solid tumour or relapsed leukaemia, chemotherapy <10 d earlier, rapid neutropaenia, cardiac and renal dysfunction	30	Positive blood culture	16	27%	0.18	1.44
Jones (1996)	Low risk = ANC ⩾ 200, outpatient at onset, in remission	127	Bacteraemia	68	17%	0.71	1.07
Lucas (1996)	Low risk = no chills, hypotension, or a requirement for fluid resuscitation at admission	509	Positive blood culture	82	87%	0.72	4.05
Petrelli (1991)	Low risk: patients with solid tumours and lymphomas stages I and II. High risk: patients with leukaemias and lymphomas stages III and IV,	146	Positive blood culture	35	45%	0.58	1.42
Rondinelli (2006)	Low risk = 2.5–5 points: Intermediate risk = 5.5–9 points: High risk = greater than 9 points 4.5 points for: clinical site of infection; 2.5 points for: no URTI; 2 points for: CVC; 1 point for: aged ⩽5 years, fever >38.5 °C, Hb ⩽7 g/dL	283	‘Serious infectious complication’ – sepsis, shock, +ve blood cultures, infection-related death	93	Odds ratio only:	Low 1.0	
						Mid 13	
						High 50	
West (2004) (bootstrapped)	High risk = temp > 39.5 °C and CRT > 3 s; Mid risk = temp > 39.5 °C or CRT > 3s; Low risk = neither	143	Requirement for critical care within 24 h of presentation (fluid boluses ⩾ 60 ml/kg, inotropes or ventilation)	36	Low 89%	0.73	Infinite
					Mid 10%	2.70	

Models with >1 supporting dataset							
*Santolaya rule*							
	Clinical prediction rule	Number in study	Outcome	Number with Outcome	% Low	LR Low	LR High

Santolaya (2001)	Low risk = 0 factors or isolated low plts or <7 d from chemotherapy. High risk = >1 risk factor, or isolated high CRP, hypotension or relapsed leukaemia. Risk factors: CRP ⩾ 90, hypotension, relapsed leukaemia, plts ⩽ 50, chemotherapy within 7 d	407	Invasive bacterial infection = positive blood culture (2 for Coagulase-negative *Staphylococcus* spp), positive bacterial culture from usually sterile site, or sepsis syndrome and/or focal organ involvement and haemodynamic instability and severe malaise	178	42%	0.22	2.41
Santolaya (2002)	As above	263	As above	140	40%	0.11	3.91

*Rackoff dichotomous rule*							
Rackoff (1996)							
(proposed from validation set)	Low risk = AMC > 100; High risk = AMC < 100	57	Bacteraemia	10	23%	0	1.45
Baorto (2001)	As above	1171	Bacteraemia	189	21%	0.45	1.45
Tezcan (2006)	As above	671	Microbiological documented infection	225	58%	0.70	1.60

	Clinical prediction rule	Number in study	Outcome	Number with Outcome	% Low	% Mid	LR Low	LR Mid	LR High
*Paganini rule*									
Paganini (2007)	Low risk < 4. Mid risk = 4. High risk = >4. Advanced stage of disease = 3 points, Comorbidity = 2 points, Bacteraemia = 1 point		Death	18	82%	10%	0	2.38	12.0
(validation set)	As above	806	Death	19	82%	12%	0.12	2.76	9.86
*Rackoff three-category rule*									
Rackoff (1996)									
(derivation set)	Low risk = AMC > 100; Mid risk = AMC < 100, and temp < 39; High risk = AMC < 100, but temp ⩾ 39	115	Bacteraemia	24	17%	65%	0	0.87	3.44
(validation set)	As above	57	Bacteraemia	10	23%	40%	0	0.21	3.52
Klaassen (2000)	As above	226	Bacteraemia	28	37%	37%	0.35	0.75	2.57
			Significant bacterial infection	43			0.39	0.94	2.29
(validation set)	As above	136	Bacteraemia	28	42%	33%	0.21	4.34	3.09
			Significant bacterial infection	26			0.59	1.28	1.41
Madsen (2002)	As above	157	Bacteraemia	12	25%	64%	0.31	0.91	3.72

ALL, acute lymphoblastic leukaemia; AML, acute myeloid leukaemia; AMC, absolute monocyte count; CoNS, coagulase-negative *Staphylococcus*; CRP, C-reactive protein; CRT, capillary refill time; CXR, chest X-ray; Hb, haemoglobin. Plt, platelet.

## Appendix A Supplementary data

Supplementary data 1 Web Appendix.
