# Supplementary material for: Systematic review and meta-analysis of the discriminatory performance of risk prediction rules in febrile neutropaenic episodes in children and young people
Source: Eur J Cancer. 2010 Nov;46(16):2950–64. doi: 10.1016/j.ejca.2010.05.024 (PMC2981857; doi:10.1016/j.ejca.2010.05.024)
Supplement: Supplementary data 1 — Web Appendix. [file mmc1.doc]

*Web Figure 1. ROC plots showing study accuracy by outcome type*

The ‘cross hairs’ ROC space plots show each study estimates of sensitivity and specificity as a marker at the point estimate, with 95% confidence intervals demonstrated by lines. In reading such graphs, tests with a better discriminatory ability fall in the top left corner of the plot, and non-discriminatory tests fall on a 45^o^ line between the bottom left and top right.

**Web Appendix 1. Search Strategy**

Example based on OVID-Medline: was adapted for other databases

*FNP identification*

1 Neutropenia/

2 (neutropenia or neutropenic).ti,ab.

3 1 or 2

4 Fever/

5 (fever$ or febril$).ti,ab.

6 4 or 5

7 3 and 6

*Child identification*

8 adolescent/ or child/ or child, preschool/ or infant/ or infant, newborn/ or Puberty/

9 schools/ or schools, nursery/

10 (infan$ or newborn$ or new born$ or baby$ or babies or neonat$ or neonat$ or child$ or schoolchild$ or kid or kids or toddler$ or adoles$ or teen$ or boy$ or girl$ or minor$ or underage$ or under age$ or juvenil$ or youth$ or kindergar$ or nursery or puber$ or prepuber$ or pre puber$ or pubescen$ or prepubescen$ or pre pubescen$ or pediatric$ or paediatric$ or peadiatric$ or school or schools or preschool$ or pre school$ or schoolage$).ti,ab.

11 8 or 9 or 10

*Cancer identification*

12 exp Neoplasms/

13 (cancer$ or neoplas$ or oncolog$ or malignan$ or tumo?r$ or sarcoma$

or leukaemi$ or leukemi$ or chemotherap$).ti,ab.

14 12 or 13

*Consolidation*

15 11 and 14

16 7 and 15

*CDR Hedge*

17 (predict$ or clinical$ or outcome$ or risk$).mp.

*Final search*

18 16 and 17

**Web Appendix 2. Modified QUADAS criteria for quality assessment.**

|  | Item | Yes | No | Unclear |
| --- | --- | --- | --- | --- |
| 1. | Was the spectrum of patients representative of the patients who will receive the test in practice? | ( ) | ( ) | ( ) |
| 2. | Were selection criteria clearly described? | ( ) | ( ) | ( ) |
| 3. | Is the reference standard likely to correctly classify the target condition? | ( ) | ( ) | ( ) |
| ~~4.~~ | ~~Is the time period between reference standard and index test short enough to be reasonably sure that the target condition did not change between the two tests?~~ | ~~( )~~ | ~~( )~~ | ~~( )~~ |
| 5. | Did the whole sample or a random selection of the sample, receive verification using a reference standard of diagnosis? | ( ) | ( ) | ( ) |
| 6. | Did patients receive the same reference standard regardless of the index test result? | ( ) | ( ) | ( ) |
| 7. | Was the reference standard independent of the index test (i.e. the index test did not form part of the reference standard)? | ( ) | ( ) | ( ) |
| 8. | Was the execution of the index test described in sufficient detail to permit replication of the test? | ( ) | ( ) | ( ) |
| 9. | Was the execution of the reference standard described in sufficient detail to permit its replication? | ( ) | ( ) | ( ) |
| 10. | Were the index test results interpreted without knowledge of the results of the reference standard? | ( ) | ( ) | ( ) |
| 11. | Were the reference standard results interpreted without knowledge of the results of the index test? | ( ) | ( ) | ( ) |
| 12. | Were the same clinical data available when test results were interpreted as would be available when the test is used in practice? | ( ) | ( ) | ( ) |
| ~~13.~~ | ~~Were uninterpretable/ intermediate test results reported?~~ | ~~( )~~ | ~~( )~~ | ~~( )~~ |
| ~~14.~~ | ~~Were withdrawals from the study explained?~~ | ~~( )~~ | ~~( )~~ | ~~( )~~ |

Note: The three criteria shown ~~struck though~~ were not used in the assessment

***Web Appendix 3. Informative QUADAS measures***

| **Citation** | **Study design** | **Verification procedure biases** | | | **Interpretation biases** | |
| --- | --- | --- | --- | --- | --- | --- |
|  |  | **Partial verification** | **Differential verification** | **Incorporation bias** | **Review bias** | **Review bias** |
|  | **Prospective or retrospective?** | **Did the whole sample or a random selection of the sample, receive adequate outcome assessment?** | **Did patients receive the same outcome assessment regardless of the CDR result?** | **Was the outcome assessment independent of the CDR?** | **Were the CDR results interpreted without knowledge of the results of the outcome assessment?** | **Were the outcome assessment results interpreted without knowledge of the results of the CDR?** |
| Adcock 1999 | Retrospective | Yes | Yes, although some tests were undertaken if clinically indicated | Yes | Unclear – not blinded | Unclear - not stated |
| Alexander 2002 | Retrospective | Not stated | Not stated | No – serious medical complication included hypotension & mucositis (which are part of the CDR) | Yes | Yes |
| Ammann, 2003 & 2004 | Retrospective | Yes | Yes, although some tests were undertaken if clinically indicated | No – one variable from CDR was in outcome assessment (C-reactive protein level, although cutpoint differed; 50mg/L in CDR vs 150mg/L in outcome assessment). | Unclear - not blinded | Unclear - not blinded |
| Baorto, 2001 | Retrospective | Yes | Yes, although some tests were undertaken if clinically indicated | Yes | Yes | Unclear - not blinded |
| Gala-Peralta, 2005 | Retrospective | Yes | Yes | Yes | Unclear – not blinded | Unclear - not blinded |
| Hann 1997 | Retrospective (RCT trial data) | Yes | Yes, although some tests were undertaken if clinically indicated | Yes | Yes | Unclear - not blinded |
| Jones 1996 | Prospective | Yes | Yes, although some tests were undertaken if clinically indicated | Yes | Yes | Unclear - not stated |
| Klaassen, 2000 | Prospective | Yes | Yes, although some tests were undertaken if clinically indicated | Yes | Yes | Yes - blinded |
| Lucas, 1996 | Retrospective | Yes | Yes | Yes | Yes | Unclear - not blinded |
| Madsen, 2002 | Retrospective electronic record | Yes | Yes | Yes | Yes | Unclear. Review was blinded, but of unblinded case notes |
| Paganini 2007 | Prospective | Yes | Yes | Yes | Yes | No - but mortality |
| Petrelli, 1991 | Prospective | Unclear | Yes | Yes | Yes | No |
| Rackoff, 1996 | Prospective (Derive) and Retrospective (Validate) | Yes | Yes | Yes | Yes (Derive) and  Unclear – not blinded (Validate) | Unclear - not blinded |
| Riikonen 1993 | Prospective | Yes | Yes | Yes | Yes | No |
| Rojo, 2008 | Retrospective | Yes | Yes | Yes | Unclear - not blinded | Unclear - not blinded |
| Rondinelli, 2006 | Retrospective | Unclear | Yes | Yes | Yes | Yes |
| Santolaya, 2001 | Prospective | Yes | Yes, although some tests were undertaken if clinically indicated | Yes | Yes | Yes - blinded |
| Santolaya, 2002 | Prospective | Yes | Yes, although some tests were undertaken if clinically indicated | Yes | Yes | Yes - blinded |
| Tezcan 2006 | Retrospective | Yes | Yes | Yes | Yes | Unclear - not stated |
| West, 2004 | Retrospective | Yes | Yes | Yes | Unclear - not blinded | Unclear - not blinded |

**Web Appendix 4. Numerical aspects of derivation studies.**

| **Citation** | **n**  **patients** | **n episodes** | **n events** | **n variables examined** | **events per var** | **All candidate variables initially examined** | **How were candidate variables selected?** |
| --- | --- | --- | --- | --- | --- | --- | --- |
| Adcock 1999 | 33 | 88 | 16 | 14 | 1.14 | Demographics, primary diagnosis, history of present illness, vital signs, and physical examination. Recent chemotherapy regimen, prophylactic (antibiotic) therapy, leukocyte count and ANC, maximum daily temperature, age, and condition of central line | Not stated |
| Alexander 2002 | 104 | 104 | 13 | 2 | 6.50 | Anticipated neutropenia <7 days, no significant comorbidity at presentation (defined later). | Literature review. |
| Ammann, 2003  (models #1 - #3) | 111 | 285 | 90 | 39 | 2.31 | 39 variables: age, gender, pre-B-cell leukaemia or other diagnosis, first or later malignancy, relapsed or unrelapsed malignancy, history of episodes of FN without significant bacterial infection, history of episodes of FN with significant bacterial infection, history of episodes of FN with bacteraemia, remission status of malignancy, bone marrow involvement, maintenance therapy or more intensive chemotherapy, delay since last chemotherapy, time since diagnosis, year of previous episode(s) of FN, season of previous episode(s) of FN, preventive application of G-CSF, central venous catheter present, hospitalisation history before FN, presence of comorbidity requiring hospitalisation, iatrogenic reason for fever, fever rule (≥38.5°C persisting for at least 2 hours or once ≥39°C), weight loss since last chemotherapy, BMI, maximal fever at presentation, general appearance, presence of chills at presentation, lowest systolic BP, lowest diastolic BP, presence of oral mucositis, presence of clinical signs of viral infection, haemoglobin level, leukocyte count, neutrophil count, monocyte count, phagocyte count, thrombocyte count, serum CRP level, serum creatinine level, and serum ASAT level. | Covariates with possible relevance to severe bacterial infections and accessible to the treating physicians within the first 2 hours after fulfilment of the criteria of FN |
| Ammann, 2004 | 132 | 364 | 85 | 39 | 2.18 |
| Hann 1997 | 759 | 759 | 165 | 13 | 12.7 | Gender, underlying disease (AML, ALL, BMT, HD/NHL, CML-aplasia-blast-crisis-other, Solid tumour), disease status (induction, relapse, maintenance), IV line in situ, defined site of infection, shock, granulocyte count, period of granulocytopenia, antifungal prophylaxis, antibacterial prophylaxis, age, temperature, (log) creatinine | Not stated |
| Jones 1996 | 127 | 276 | 68 | 5 | 13.6 | Underlying disease and status (i.e. induction therapy, remission or relapse). Age at time of fever episode. ANC at time of onset of fever. Inpatient versus outpatient status | Not stated |
| Klaassen, 2000 | 140 | 227 | 28 | 13 | 2.15 | 13 variables assessed: age, presence of bone marrow disease, central venous catheter type, general appearance on initial examination, previous granulocyte colony-stimulating factor (G-CSF) therapy, initial ANC, initial lymphocyte count, initial monocyte count, initial platelet count, presence of localized bacterial infection on initial examination, peak temperature, tumour type, sex. | Systematic review to identify risk factors for significant bacterial infection and expert opinion. |
| Lucas, 1996 | 161 | 509 | 82 | 8 | 10.25 | Chills, hypotension, poor perfusion, the need for fluid resuscitation, time from cytotoxic chemotherapy, diagnosis, disease status, and the presence of a focus of infection | Not stated |
| Paganini 2007 | 458 | 714 | 18 | 17 | 1.06 | Age, days since chemotherapy, ‘advanced stage of disease’ (= bone marrow involvement, relapse, second tumour, high-dose therapy, genetic disease), previous antibiotic or CSF use, ANC <100, clinical infection, pneumonia, mucositis, bacteremia <24h, comorbidity (=incoercible bleeding, refractory hypoglycaemia and hypocalcemia, hypotension, altered mental status, renal insufficiency, hepatic dysfunction, and respiratory failure). They also state that the following variables were collected and registered for analysis: facial, anal, oral or catheter-associated cellulitis, sepsis, necrotising gingivitis, sex, underlying disease and staging, predicted period of neutropenia, presence of intravenous device. | Unclear |
| Rackoff, 1996 | 72 | 115 | 24 | 9 | 2.67 | State of disease (remission vs not), degree of mucositis, ill appearance, presence of GI symptoms, cellulitis, use of GCSF, admission ANC, admission AMC, maximum admission temperature | Unclear |
| Rackoff, 1996 revised model | 102 (see note) | 57 | 10 | 7 | 1.43 | AMC, Temperature (39.5C cutoff), ANC, APC, Platelets, age, WBC | By reference to previously published literature |
| Riikonen 1993 | 46 | 91 | 17 | 16 | 0.94 | Duration of fever, duration of neutropenia, central line present, prophylaxis with Septrin, general clinical examination, HR, signs of bleeding, BP, temperature, chills, Hb, Plt, prolonged PTT, sodium & potassium ESR, CRP | Unclear |
| Rojo, 2008 | 33 | 47 | 4 | 6 | 0.67 | Sex, age, type of malignancy (leukaemia vs solid), focus of infection, duration of hospitalisation, microbiologically proven infection | Unclear |
| Rondinelli, 2006 | 283 | 283 | 93 | 17+ | 5.47 | Significantly on univariate: Age, gender, disease type (AML, ALL, Others), disease status (remission/other), CVC, temperature, Hb, WCC, AGC, Plt, AMC, URTI, time from chemotherapy, pneumonia, clinical site of infection, mucositis plus others not reported | Unclear |
| Santolaya, 2001 | 257 | 447 | 179 | 17 | 10.5 | (1) demographic variables, ie, age, sex, and maternal educational level; (2) cancer-related variables, ie, cancer type, intensity of chemotherapy, use of granulocyte colony-stimulating factors since last administration of chemotherapy, and use of an indwelling catheter; (3) variables related to the febrile episode, ie, hours of fever before admission, days since last administration of chemotherapy, and use of prophylactic antimicrobial agents; (4) admission clinical and laboratory variables, ie, axillary temperature, blood pressure, ANC, AMC, quantitative serum CRP level, hemoglobin level, and platelet count | Not stated |
| Tezcan 2006 | 240 | 621 | 143 | 11 | 13.0 | Age, sex, ANC, AMC, CRP, duration of neutropenia, duration of fever, presence of previous FN, presence of hypotension, uncontrolled malignancy, cancer type. | Unclear |
| West, 2004 | 143 | 303 | 36 | 18 | 2.00 | Age, type of cancer, chills, temperature, HR, RR, SBP, DBP, mucositis, Hb, Plts, WCC, differential WCC, ANC, AMC, monocytes <10%, perirectal abscess, capillary refill time >3s. | Review of literature + medical opinion. |

Note: 102 minus participants excluded for meeting exclusion criteria

**Web Appendix 5. Variable and missing data handling techniques in derivation studies.**

| **Citation** | **Statistical technique to build the model** | **Management of multiple episodes.** | **Management of continuous variables…** | **… and cutpoint determination** | **Management of categorical variables.** | **and cutpoint determination** | **n patients or episodes with missing values** | **Management of missing data.** |
| --- | --- | --- | --- | --- | --- | --- | --- | --- |
| Adcock 1999 | Univariate analysis | No discrimination | Made ordinal (BP described as ‘hypotension’ or not) | Not stated | Grouped (Ara-C vs Other chemotherapy) | Based on “trend to significance” from univariate analysis | Not stated | Not stated |
| Alexander 2002 | Univariate analysis | First episode only used | Made ordinal (hypotension and hypoxia) | Not stated | Grouped (‘anticipated neutropenia’ group by cancer type – AML/Burkitts/Induction ALL/Progressive-relapsed with marrow involvement vs not) | Not stated. | 2 patients excluded due to missing data | Completed data only used |
| Ammann, 2003 | Decision tree, regression type | First-last comparison | Made ordinal, with up to three categories. | Not stated | Grouped | Not stated | 1 patient (2 episodes) excluded due to missing data. exclusion of 41 episodes where >10% of covariates were missing | Completed data only used. Covariates with more than 10% missing values were discarded from the prediction model |
| Ammann, 2004 | Stepwise backward | First-last comparison | Made ordinal, with up to three categories. | Not stated | Grouped | Not stated | 2 patient (2 episodes) excluded due to missing data. exclusion of 16 episodes where >10% covariates were missing | Completed data only used. Covariates with more than 10% missing values were discarded from the prediction model |
| Hann 1997 | Stepwise backward | First episode only used | As continuous values, or if skewed distribution, categorised according to clinical judgement | According to clinical judgement | Grouped | According to clinical judgement | Not stated (multivariate analysis was based on 678 children. For one of the included trials data were available for 145/220 children) | Not stated |
| Jones 1996 | Logistic regression - unclear | No discrimination | Made ordinal (e.g. age <2, 2-5, 6-12, 13+, ANC <200, ≥200). | Not stated. | Grouped (e.g. solid tumour, leukaemia, other). | Not stated. | Not stated | Not stated |
| Klaassen, 2000 | Forward logistic regression | Generalized linear mixed model | All continuous variables except age were dichotomised. | Using predefined thresholds taken from the literature, or recursive partitioning for monocyte count and peak temperature. | Dichotomised (tumour type – AML/NHL versus others). | According to clinical judgement | Derivative set – 1. | When monocyte count was not available the patient was excluded (n=1), for other variables it was unclear (all 13 variables were prospectively collected in 98% of the episodes). |
| Lucas, 1996 | Logistic regression - unclear | GEE | Made ordinal (time from chemo: ><10d, ANC ><100/mm) | Not stated. | Grouped | Not stated | Unclear | Not specified |
| Paganini 2007 | Forward logistic regression | No discrimination | Made ordinal (e.g. ANC <100) or used as continuous (e.g. age & days since chemo) | Not stated. | Not used |  | Not stated | Not stated |
| Rackoff, 1996 | Backwards logistic regression | GEE | Initially continuous, | then recursive partioning analysis | Grouped – disease state into remission vs relapse/progressive | Not stated | D: One patient had missing diff WBC for one episode – excluded leaving 115 episodes | Completed data only |
| Rackoff, 1996 revised model | Logistic regression - unclear | No discrimination | Made ordinal (dichotomous) | The AMC, APC and ANC cut-off values of 250/mm3 and temperature of 39.5C were selected arbitrarily. AMC cut off of 100/mm3 was used due to previous study findings (Rackoff, 1996). Serial NLR determined at intervals of 5 units/mm3 across the range of AMC, ANC and APC values. Platelet count values >25,000/mm3 were tested at intervals of 25,000/mm3. | Not applicable |  | Validation set – 82 (60%) episodes | Not stated |
| Riikonen 1993 | Univariable analysis | No discrimination | Made ordinal: Hb <100g/l, Plts <10, 10-30, 30-100), PT ‘prolonged’, Na & K: less than normal limits. Kept continuous: ESR & CRP | Not stated | Not applicable |  | Unclear. | Not stated |
| Rojo, 2008 | Univariable analysis | No discrimination | Not applicable |  | Grouping (solid vs haematological malignancy) | Not stated | Not stated | Not stated |
| Rondinelli, 2006 | Forward logistic regression | First episode only used | Made ordinal | Not stated, but use previously defined cut-offs | Grouped | Not stated | Not stated | Completed data only |
| Santolaya, 2001 | Forward logistic regression | Secondary analysis undertaken with first episode only | As continuous values initially | Then cutpoints determined with ROC (for CRP and platelets) | Grouped (ALL, AML, lymphoma, sarcoma, relapsed leukaemia, other solid) | Not stated | Not stated | Not stated |
| Tezcan 2006 | Logistic regression - unclear | No discrimination | Some kept continuous (e.g. age, CRP, duration of fever, duration of neutropenia), some made ordinal (e.g. ANC <100, AMC <100). | Not stated | Grouped (e.g. cancer type: leukaemia and lymphoma vs solid tumours | Not stated | Not stated – different outcome categories have different total values | Not stated |
| West, 2004 | Stepwise backward & bootstrapping | Multivariate analysis "adjusted for clustering at patient level" | Some continuous (temp, age, heart rate z-score) some categorised (BP dichotomised to -2SD, monocytes <10% and ANC=0) | Not clearly described | Categorised: type of cancer – leukaemia/lymphoma, sarcoma/neuroblastoma, other | Not stated | Not stated | Not stated |

**Web Appendix 6. Individual factors used in clinical prediction rules**

| **Variable** |  | **Citation** | **Citation** | **Citation** | **Citation** | **Citation** | **Citation** | **Citation** |
| --- | --- | --- | --- | --- | --- | --- | --- | --- |
| ***Pt related*** |  |  |  |  |  |  |  |  |
| **Age** |  |  |  |  |  |  |  |  |
|  | **< 6yr** | Amm03 | (Madsen) | (Tezcan) |  |  |  |  |
|  | **< 5yr** | Rondinelli |  |  |  |  |  |  |
| **Disease** |  |  |  |  |  |  |  |  |
|  | **AML/Burkitts/Induction ALL/Relapse-progressive/BM involvement** | Alexander |  |  |  |  |  |  |
|  | **Leuk/Lymph or BMT vs Other** | Hann | (Lucas) |  |  |  |  |  |
|  | **BM involved** | Amm03 | Amm04 | (Klassen) |  |  |  |  |
|  | **Pre-B Leukemia** | Amm03 |  |  |  |  |  |  |
|  | **In PR/CR** | Amm04 | (Rondinelli) | (Tezcan) |  |  |  |  |
|  | **Induction/relapse vs. remission** | Jones |  |  |  |  |  |  |
|  | **Advanced disease** | Paganini |  |  |  |  |  |  |
|  | **Relapsed leuk** | Santolaya |  |  |  |  |  |  |
| ***Rx related*** |  |  |  |  |  |  |  |  |
| **Type of Rx** |  |  |  |  |  |  |  |  |
|  | **Ara-C < 7d** | Adcock |  |  |  |  |  |  |
|  | **Low intensity (anticipated neutropenia <7d)** | Alexander |  |  |  |  |  |  |
|  | **Chemo <7d** | Santolaya |  |  |  |  |  |  |
| **Others** |  |  |  |  |  |  |  |  |
|  | **CVC present** | Amm03 | Hann | Rondinelli |  |  |  |  |
| ***Episode related*** |  |  |  |  |  |  |  |  |
| **Airway/Breathing** |  |  |  |  |  |  |  |  |
|  | **Tachypnoea/hypoxia** | Alexander |  |  |  |  |  |  |
| **Circulation** |  |  |  |  |  |  |  |  |
|  | **Hypotension** | Adcock | Santolaya | (Tezcan) |  |  |  |  |
|  | **Chills/hypotension/fluid bolus composite** | Lucas |  |  |  |  |  |  |
|  | **Shock** | Hann |  |  |  |  |  |  |
|  | **CRT >3** | West |  |  |  |  |  |  |
| **Neurology** |  |  |  |  |  |  |  |  |
|  | **Altered mental status** | Alexander |  |  |  |  |  |  |
|  |  |  |  |  |  |  |  |  |
| **Source** |  |  |  |  |  |  |  |  |
|  | **Inflammed CVC site** | Adcock |  |  |  |  |  |  |
|  | **CXR +ve / pneumonia** | Alexander | (Rondinelli) |  |  |  |  |  |
|  | **Mucositis** | Alexander | (Rondinelli) |  |  |  |  |  |
|  | **Vomiting/diarrhoea** | Alexander |  |  |  |  |  |  |
|  | **Viral infection** | Amm03 | Rondinelli (no URTI) |  |  |  |  |  |
|  | **Known bactermia** | Paganini |  |  |  |  |  |  |
|  | **Clincal site** | Rondinelli | (Madsen) |  |  |  |  |  |
| **Temperature** |  |  |  |  |  |  |  |  |
|  | **Cont variable** | Hann | Rackoff | Madsen | West |  |  |  |
|  | **<38.5** | Rondinelli |  |  |  |  |  |  |
|  | **<39.7 C** | Amm04 |  |  |  |  |  |  |
|  | **<39 (also 39.5 used)** | Klassen | (Santolaya) |  |  |  |  |  |
| **Others** |  |  |  |  |  |  |  |  |
|  | **Other comorbidity** | Alexander | Amm04 | Paganini |  |  |  |  |
|  | **Abdominal pain** | Alexander |  |  |  |  |  |  |
|  | **OP at start** | Jones |  |  |  |  |  |  |
| ***Blood work*** |  |  |  |  |  |  |  |  |
| **FBC** |  |  |  |  |  |  |  |  |
|  | **WCC >0.5** | Amm03 | Amm04 | (Madsen) | (Rondinelli) |  |  |  |
|  | **Hb <7** | Rondinelli | Amm03 |  |  |  |  |  |
|  | **Granulocytopenia >15d** | Hann |  |  |  |  |  |  |
|  | **ANC** | Jones | (Rackoff) | (Lucas) | (Madsen) | (Tezcan) | (West) |  |
|  | **AMC** | Klassen | Rackoff | (Paganini | (Santolaya) | (Baorto) | Madsen | (Tezcan) |
|  | **Plts** | Santolaya | (Madsen) | (Rondinelli) |  |  |  |  |
| **Biochem** |  |  |  |  |  |  |  |  |
|  | **CRP >50** | Amm03 |  |  |  |  |  |  |
|  | **CRP >90** | Santolaya |  |  |  |  |  |  |

Study citations in (brackets) refer to those assessed but not included in models.
